# Health Tract No. 343

**Published:** 1868-11

**Authors:** 


HEALTH TRACT NO. 343.

### FOOD CURE.

A glass of water quenches the thirst of the infant and of the pilgrim of four-score, and is as sweet and efficacious at the second as in the first childhood. A loaf of bread satisfies hunger to-day; it will a hundred years to come; these are nature's remedies for hunger and thirst: but, if a man has a thirst for rum, or brandy, or whiskey, or craves opium, he must take more a year hence to produce a specific effect, than he does to-day; a tablespoonful of brandy has a less and less stimulating effect, so with tea and coffee, as all who use them know: but a given amount of water or of bread will satisfy thirst and hunger for years together, and this should make the distinction between natural and healthful remedies, and artificial and unhealthful remedies; whatever requires to be taken oftener, or in larger quantities, to produce a specific effect, ought to be discarded as soon as possible.

Some few kinds of food have all the necessary ingredients of healthful nutriment and life, and health can be sustained for years together by living on them exclusively; while if a man is confined to one single article of diet, he will become diseased and die; simply, because he has not some ingredient which is necessary to the health of the system; hence close medical observers have ascertained that if a man has certain symptoms of disease, it arises from the fact, that the food which he has been eating has been deficient in certain ingredients, and the mode of cure is to furnish food which has these ingredients abundantly; this is, properly speaking, the "Food cure." Diarrhœa is often cured by quiet, warmth and boiled rice, because rice is "binding;" constipation may be obviated by using ripe fruits or berries largely, because they are "loosening." If the face is pale or "bloodless" or of a greenish tinge,—as the "green sickness" or CHLOROSIS—it is because there is a want of iron in the blood, for iron gives the blood its color; and CHALYBIATES or water containing iron should be used. Sometimes the bones are so soft they bend and the person can scarcely stand, this is being "rickety;" so, also, in consumption and scrofula, there is not lime enough in the system to harden the bones and give ability to exercise; then food must be eaten which has a great deal of lime, as also phosphorus, such as coarse wheaten bread and eggs and fish. Persons soon get scurvy who live on salt provisions; but get well immediately on eating vegetables; because vegetables have POTASH in them, that is, if burned to ashes, these ashes make ley, which, if boiled, leaves a sediment, which is potash or saleratus. If we eat no table salt, we shall have worms. So the time may come when we shall know enough to prevent and cure disease by natural agencies; by the food we eat; by the aid of the butcher and green grocer, and then the apothecaries will starve to death; then will come the Hygienic Millenium.

